# Odor Uniformity among Tomato Individuals in Response to Herbivore Depends on Insect Species

**DOI:** 10.1371/journal.pone.0077199

**Published:** 2013-10-09

**Authors:** Alicia Bautista-Lozada, Francisco Javier Espinosa-García

**Affiliations:** 1 Posgrado en Ciencias Biológicas, Centro de Investigaciones en Ecosistemas, Universidad Nacional Autónoma de México, Morelia, Michoacán, México; 2 Laboratorio de Ecología Química, Centro de Investigaciones en Ecosistemas, Universidad Nacional Autónoma de México, Morelia, Michoacán, México; Centro de Investigación y de Estudios Avanzados, Mexico

## Abstract

Plants produce specific volatile organic compound (VOC) blends in response to herbivory. Herbivore-induced blends may prime the plant for future attack or attract carnivorous insects; these responses have been considered adaptive for plants. If herbivores differentially modify the VOC emission among individuals within a group of plants they feed upon, then plant responses to herbivores will not only produce specific blends but also variation in odor among individuals, i.e. individuals smell the same, then having a uniform odor. We investigated the VOC emission variation or uniformity among tomato individuals (*Solanum lycopersicum* L. cv. Castlemart) in response to moderate wounding by (1) nymphs of the psyllid *Bactericera cockerelli* (Sulc.) (TP); (2) Lepidoptera chewing-feeding larvae of Fall Armyworm (*Spodoptera frugiperda* Smith) (FAW) and (3) of Cabbage Looper (*Trichoplusia ni* Hübner) (CL), and (4) mechanical damage (MD). We used a ratio-based analysis to compare the fold-change in concentration from constitutive to induced VOC emission. We also used size and shape analysis to compare the emission of damaged and non-damaged individuals. Aside of finding herbivore-specific blends in line with other studies, we found patterns not described previously. We detected constitutive and induced odor variation among individuals attacked by the same herbivore, with the induced odor uniformity depending on the herbivore identity. We also showed that the fold-change of VOCs from constitutive to induced state differed among individuals independently of the uniformity of the blends before herbivore attack. We discuss our findings in the context of the ecological roles of VOCs in plant-plant and plant-carnivore insects’ interactions.

## Introduction

The presence of insects in a field can be affected by plant emission of volatile organic compounds (VOCs) that constitute plant odor [1]. The VOC blend changes in response to herbivore attack [2,3] thus, plant emissions are considered plastic [4]. The phenotypic variation in the VOC emission among individuals in response to insects may be important in plant interactions with the third trophic level [5,6] because insect herbivory may signal the presence of the herbivore to their insect predators [7]; thus herbivore induced VOC emissions have been considered adaptive for plants. In addition, herbivore-induced VOCs may activate a systemic resistance in the nearby leaves priming the plant for a possible future attack [8].

Previous comparative studies on the chemical changes induced by different herbivores have found herbivore-specific blends in crop species such as tomatoes [9–12]. These studies compared the effect of damage on the total VOC amount, and in blend composition between damaged and non-damaged groups of plants. Composition is determined by the absolute and relative VOC concentration in the blend, indicating which VOCs dominate and contribute to plant odor, respectively [13,14]. However, mean comparisons between groups of damaged and non-damaged plants do not take into account that the constitutive and induced emissions can vary among individuals. In other words, the variation in the VOC emission among individuals is only observed as the variance around the mean.

The most common method to measure herbivore-specific responses in plants is the mean and variance comparison of a control group (non-damaged individuals) and groups of plants subjected to herbivory. This approach is perfectly correct to detect differences among treatments but fails to detect the phenotypic variation in VOC emission of individuals due to constitutive variation. Changes in the VOC emission may be undetected in the first stages of insect infestation when herbivores can feed intermittently. Continuous damage during insect feeding can maximize VOC emissions [15]. Thus, changes in the VOC emission in response to moderate damage are at risk of not being detected because insects may walk, feed on plant tissue and then leave [16]. However, little changes in the VOC emission could start behavioral responses in the biotic environment [8].

It bears consideration that despite the above-cited studies demonstrating herbivore-specific VOC blends, research has still yielded little information as to how different herbivores affect the phenotypic variation among tomato individuals within a group. Plants can differentially respond to herbivore identity in terms of the individual VOC concentration and at the whole VOC blend level. Thus, an alternative approach is called for in order to expand the understanding of the VOC variation induced by specific herbivores. Hence, a measure of the phenotypic variation among individuals damaged by the same agent is included in the analysis.

Detection of individual plant changes in the VOC emission to moderate herbivory is relevant to understand plastic responses to different intensities of damage. The measurement of changes in the VOC phenotypic plasticity based on ratios (i.e. induced VOC emission minus the constitutive emission/constitutive emission [17]) would reflect the fold-change in concentration induced by different herbivores and control for phenotypic variation. Based on the fold-change in concentration and on size and shape analysis of the absolute and relative concentration [13,14], the uniformity in odor among damaged individuals can be analyzed by calculating the phenotypic distances among individuals. The uniformity in odor reflects the similarity in the VOC emission among individuals subjected the same type of damage (artificial or biotic) which has to be analyzed not only by changes in single compounds but at the whole blend level. By producing homogeneous changes and low values of phenotypic distances in the VOC concentration among individuals, the odor of damaged plants would be uniform because all plants would transmit the same odor signal. In contrast, heterogeneous changes and high phenotypic distances among individuals would produce different odors within the group.

In this study, we analyzed the absolute and relative concentration of VOCs emitted by individual tomato plants (*Solanum lycopersicum* cv. Castlemart) before and after damage by either one of three insect herbivores *Bactericera cockerelli* (Hemiptera; Psyllidae), *Trichoplusia ni* and *Spodoptera frugiperda* (Lepidoptera; Noctuidae), or by mechanical damage. Changes in the VOC emission among individuals were analyzed using two approaches: (1) the fold-change in concentration from constitutive to induced state based on the VOC collection of the same individual before and after damage, and (2) size and shape analysis, which allows the study of the variation of the absolute and relative concentration among treatments. We found herbivore-specific VOC emissions and herbivore-specific uniformities in odor among tomato individuals.

## Materials and Methods

### Plants

Castlemart tomato seeds were donated by Dr. John Délano-Frier from CINVESTAV-IPN. Plants were grown in peat moss COSMOPEAT (Canadian sphagnum peat moss) under greenhouse conditions (28°C±4 and 60% relative humidity during spring and summer). Once germinated, plants were transplanted to 256 mL plastic pots with the same substrate and watered daily without fertilization. We used plants with three to four fully extended leaves (about 30 days old) for experiments.

**Figure 1:**
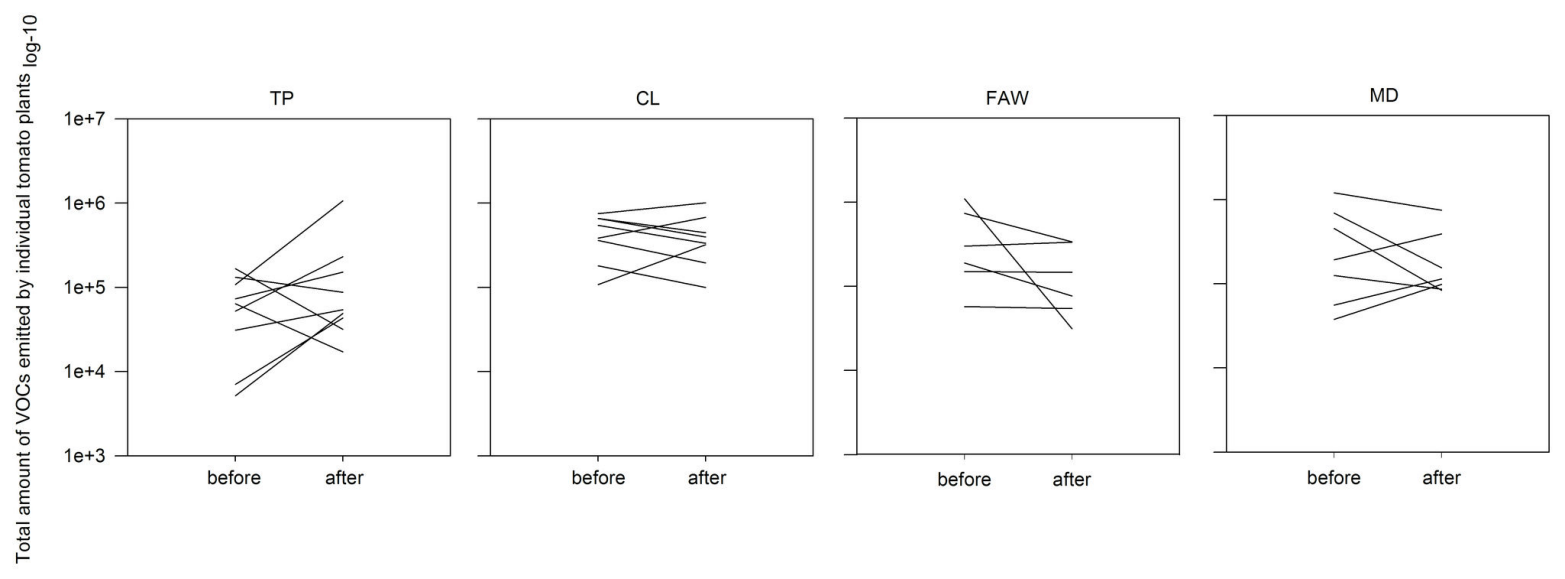
Total concentration of volatile organic compounds emitted by Castlemart tomato individuals (mean of peak areas of all compounds in the blend) before and after damage by Tomato psyllid nymphs (TP), Cabbage looper caterpillars (CL), Fall Armyworm caterpillars (FAW), and Mechanical damage (MD). Each line represents a plant within each treatment.

### Insects

Nymphs from 4th and 5th instars of tomato psyllid nymphs (TP) were obtained from the UNAM chemical ecology laboratory and reared on tomato (*Solanum lycopersicum* L.) and chili (*Capsicum annum* L.) plants under laboratory conditions (16 h light/8 h dark photoperiod at 25±3°C). Cabbage looper (CL) and Fall Armyworm (FAW) caterpillars were obtained from INIFAP (Instituto Nacional de Investigaciones Forestales Agrícolas y Pecuarias). CL caterpillars were reared on an artificial diet [18] for their complete life cycle. FAW caterpillars were reared with this diet until the third instar and then fed with castor bean leaves (*Ricinus communis* L. (Euphorbiaceae)). In our laboratory, caterpillars were only fed with castor bean leaves since hatching.

### Induction treatments

Four induction treatments were performed, with the three insect species mentioned above and with mechanical damage. The VOCs of plants were collected before and after treatments. For the TP damage treatment, six individuals from 4th and 5th instars were placed on one leaflet and were allowed to feed continuously for five days (n=9 plants). For the CL and FAW damage treatments (n=8 and 6 plants, respectively), one 3rd instar individual was placed on a leaflet and allowed to feed continuously for four hours. Mechanical damage (MD) treatment (n=8 plants) consisted of cutting the longitudinal half of one leaflet using scissors. For TP-damaged plants, VOCs were collected at day one and five because induced responses to phloem-sucking insects have been reported to start after this period [19]. Nymphs were feeding on the leaflet during VOC collection. For CL, FAW and MD treatments, VOCs were collected on day one and two after 24 hours of the damage onset. Caterpillars were removed from plants before collection. During herbivore feeding, plants were inside plastic pots covered with a fine mesh cloth under laboratory conditions, 25±3°C, 16 h light/8 h dark photoperiod with incandescent light (400-500 lumens).

### Volatile collection set up

A 2.5 L glass-collection apparatus similar to the one used by Sánchez-Hernández et al. [20] was used for VOC collection. The one used in this work was larger. This apparatus consists of two half-glass cylindrical cells, the base and the upper cell. In front of the upper glass cell of the apparatus, there is a tube with an aluminum cap and a rubber septum to allow the insertion of a solid phase microextraction (SPME) fiber (50/30 µm divinylbenzene/carboxen/polydimethylsiloxane, Supelco, Bellefonte, USA). In the upper glass cell there were two air conducts.

**Figure 2:**
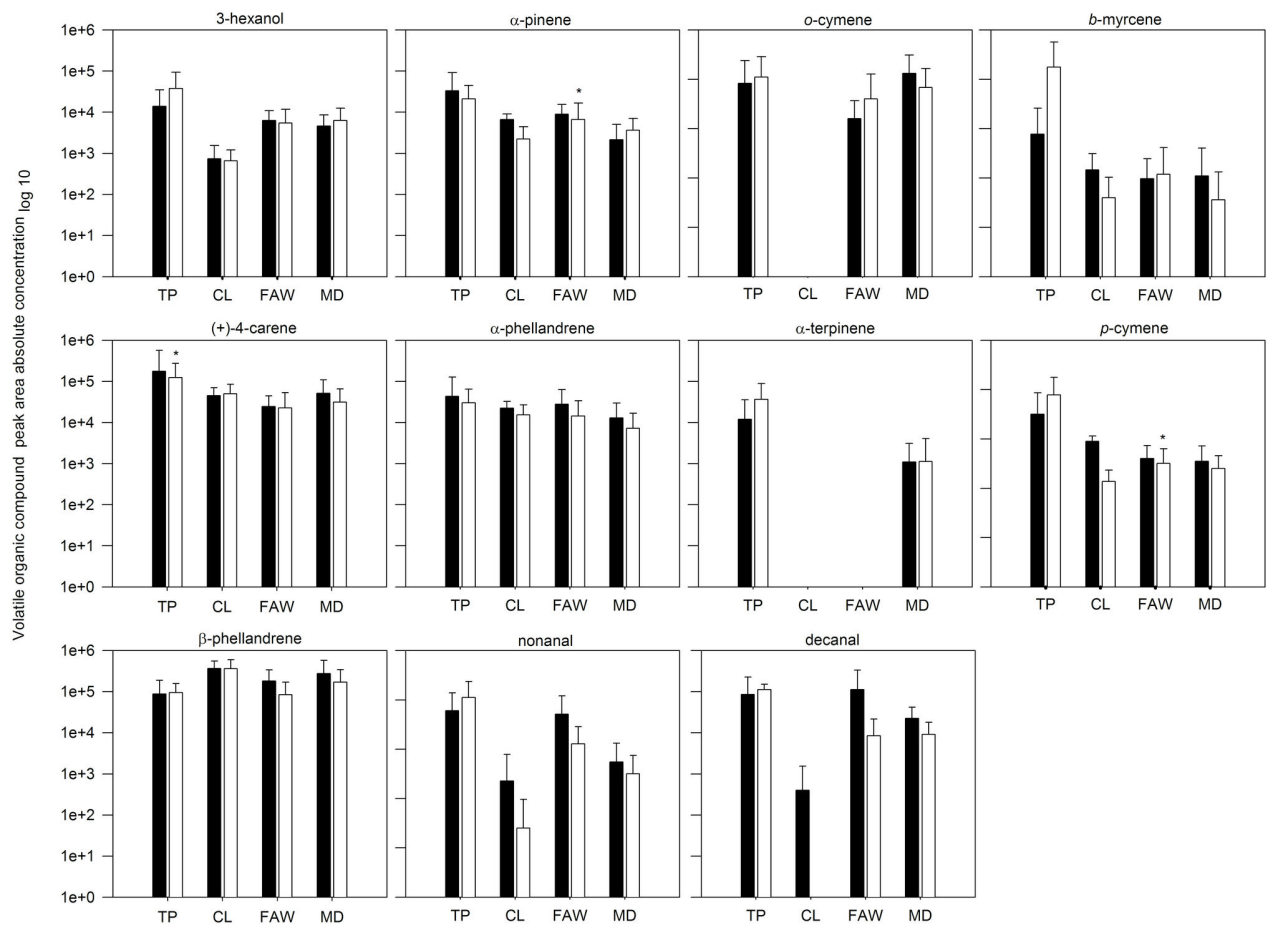
Absolute concentration (mean ± SD of peak area) of volatile organic compounds emitted by Castlemart tomato plants before (black bars) and after (white bars) damage treatments. Footnote of Figure 2. Peak absolute concentration represents the abundance of a single volatile in the blend. Damage treatments: TP, tomato psyllids (n=9); CL, Cabbage looper caterpillars (n=8); FAW, Fall armyworm caterpillars (n=6); MD, Mechanical damage (n=7). Asterisks indicate significant differences in the absolute concentration of volatile emissions before and after damage (paired-t test or Wilcoxon test at *P*=<0.05).

The plant was placed on the base part. To exclude the aerial part of the plant from the pot a split Teflon® disk was placed around the stem. Additionally, the space that was left around the stem was covered with Teflon tape. Then, the upper part was placed over the aerial part of the plant with the two air conducts open to vent possible VOC bursts due to manipulation. Plants were left under these conditions for an hour before VOC collection to let the natural airflow to stabilize them. In addition, this one-hour period was also considered a period of VOC pre-concentration for SPME collection. At the end of this period, the air conducts of the upper glass chamber were closed with Teflon tape to create a static headspace. The base and upper parts of the collection apparatus were secured with a pair of 40 mm paper clips. The exposed SPME fibers were introduced in the insertion tube and exposed for 30 min. The same fiber was used to collect the VOC emission of the same plant before and after damage.

### Volatile identification

Fibers were injected into a gas chromatograph (GC, Agilent 6890) (Capillary column Equity-1 30 m, 0.25 mm, 0.25 µm, polydimethylsyloxane, Supelco, USA) coupled with a mass spectrometer (MS) model Agilent 5973. The SPME fiber was placed in the injector at 180°C, in splitless mode (5 min) followed by split mode (5 min). GC oven temperatures started at 42°C, which was held for 3 minutes and then raised 1.5 °C/min until they reached 55°C, then raised 3 °C/min until they reached 120°C, which was held for 5 minutes (modified from [20]). The GC-MS transfer line was set at 280°C and the ionization source at 70 eV. Compounds were identified by comparing their spectra with those of the US National Institute of Standards and Technology 98 library and calculating Kovats indexes (KI) by comparing VOC retention times with the retention time of a C8-C20 series of n-alcanes (obtained under the same conditions used for plant VOCs). Standards for β-myrcene and nonanal (Aldrich), and α-pinene, α-phellandrene, and decanal (Sigma) were injected for further identification confirmation. The absolute concentration of individual VOCs was calculated from their peak areas. Total VOC emissions were estimated by the sum of the absolute concentration of each compound in the blend. Then, the relative concentration of each compound was calculated from the sum of the peak areas of all compounds in the blend.

**Figure 3:**
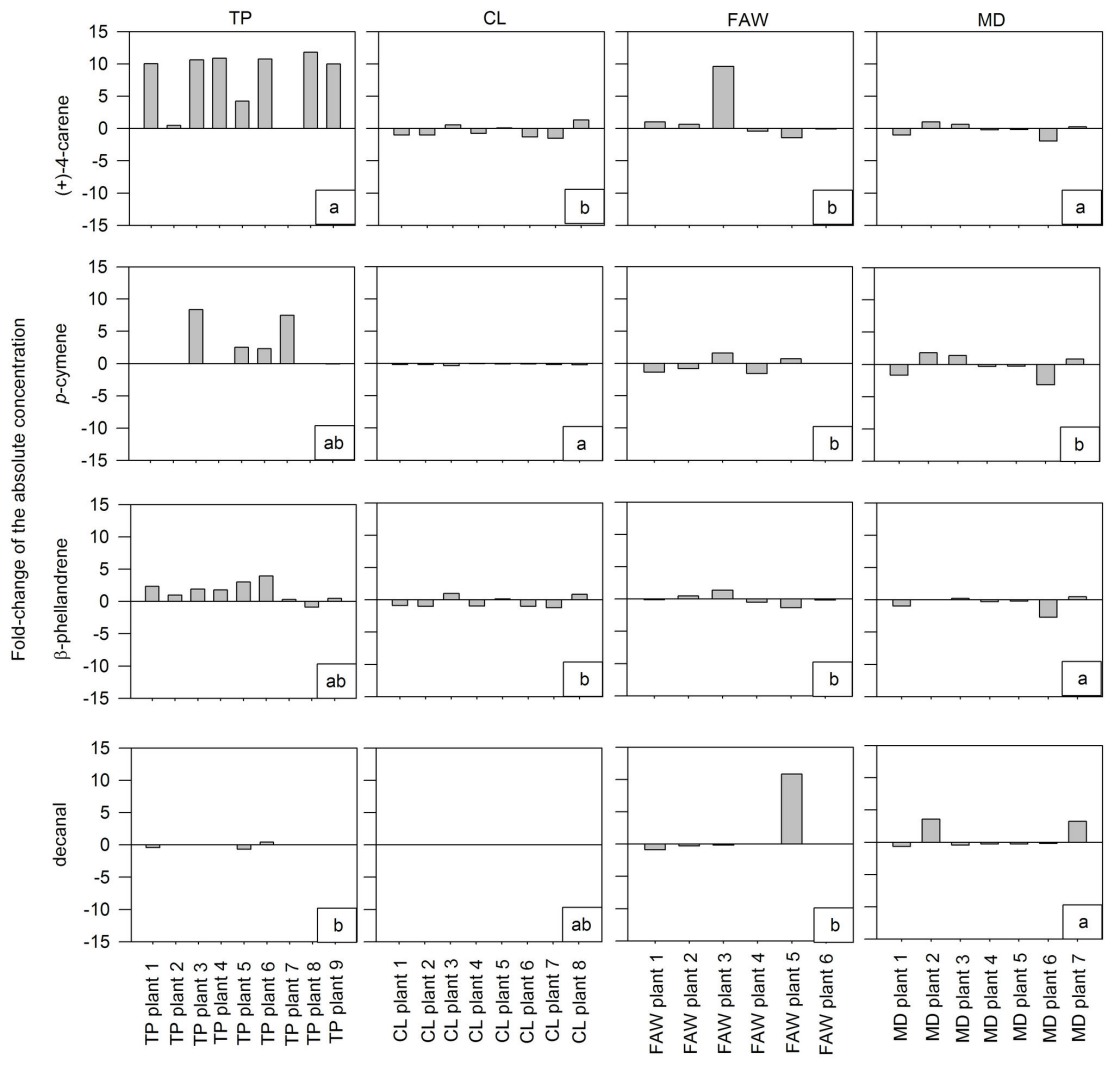
Volatile organic compounds absolute concentration fold-change from the constitutive to the induced state emitted by Castlemart tomato plants under different damage treatments. Footnote of Figure 3. The absolute concentration is the abundance of single volatiles in the blend calculated from peak areas in chromatograms. Damage treatments: TP, tomato psyllids; CL, Cabbage looper caterpillar; FAW, Fall armyworm caterpillars; MD, Mechanical damage. Bars represent the fold-change of individual tomato plants within treatments. Spaces without bars indicate lack of change or a fold-changes smaller than 1 (see Table S1).

### Statistical analysis

Based on peak abundances of each compounds we determined the absolute concentration of VOCs. Then, the relative concentration was determined as the proportion of each compound in the blend. To analyze the effect of different damage treatments over the VOC emission composition (the absolute and relative VOC concentration) of tomato individuals, we used two approaches: the fold-change in concentration from the constitutive to the induced state and size and shape analysis.

The fold-change in concentration (Δ) is a ratio based on the concentration of VOCs before and after damage. We calculated this ratio using the formula Δ= (T_2_ - T_1_)/T_1_ where T_1_ is the VOC concentration at time one (i.e., before damage) and T_2_ is the VOC concentration at time two (i.e., after damage) [17]. Using the fold-change in concentration we compared chemical differences with respect to each plant’s own constitutive emission.

To perform size and shape analysis, the peak area from VOCs detected in the blend of the 30 plants before damage was pooled to be analyzed as the control group. Size and shape analysis allowed us to study differences in the absolute and relative VOC concentration of individual secondary metabolites, respectively [13,14]. The variable size was calculated by the log_e_+1 transformation of VOCs peak areas. The subtraction of the geometric mean from size values produced the shape variable. The geometric mean was calculated from the log_e_+1 transformation of all VOCs for each plant treatment. Hexanal was excluded from the size analysis because it showed little variation among tomato genotypes and high negative correlation to nonanal (r=0.59, *P*=<0.001). The rank of the matrix of the shape variable is one less than that of size matrix [13], thus we excluded variable log_e_+1(α-terpinene)- log_e_(geometric mean) from the analysis. α-terpinene showed little variation and its coefficient value in the linear discriminants did not significantly contributed to the discrimination of damaged plants.

Fold-change in concentration and size-and-shape matrices were analyzed by a multivariate analysis of variance (MANOVA). For the fold-change MANOVA, we compared ratio values of individual compounds using damaged treatments as categorical variables. For size and shape MANOVA, data from non-damaged (ND) and damaged treatments (TP, FAW, CL, and MD) were used as categorical variables. MANOVA analyzes were followed by a one-way analysis of variance (ANOVA) if we detected a *P* value lower than 0.05 for the absolute or relative concentration. In addition, we conducted a paired t-test using log-transformed data to detect changes in the absolute and relative concentration of VOCs before and after damage treatments.

To analyze the uniformity in odor among individuals within each damage treatment, we performed a linear discriminant analysis. Then we calculated the phenotypic distances (Euclidian distances) among individuals from coordinates derived from each linear discriminant (LD). The ordination LDs obtained from both analyses that accounted for at least 85% of the variation was used to calculate the phenotypic distances among individuals. The phenotypic distances that resulted from the constitutive and induced size-and-shape analysis were used to analyze differences in the blend of individuals within and among treatments, while those that resulted from the fold-change analysis were used to analyze variation of plastic responses to each treatment. To compare the mean phenotypic distances among treatments we used an ANOVA or a Kruskal-Wallis test. R software version 2.14.1 was used to perform all statistical analyses.

**Table 1:** MANOVA and Linear Discriminant Analysis (LDA) for fold-change in concentration from constitutive to induced volatile organic compounds (VOCs) emission (A) and size and shape VOC concentration analysis (B) for Castlemart tomato plants under different damage treatments.

		**MANOVA**				**LDA**							
						**Eigenvalues**					**Cumulative variation**		
**VOCs**	**Concentration**	**d.f.**	**Pillai**	***F***	***P***	**LD1**	**LD2**		**LD3**		**LD1**	**LD2**	**LD3**
A. Fold-change	Absolute	3	1.6837	2.093	<0.001	5.79		4.28		1.52	40	72	89
	Residuals	26											
	Relative	3	2.1319	4.0186	<0.001	7.48		5.36		2.92	60	91	
	Residuals	26											
B. Size and Shape	Absolute	4	1.3171	2.1423	<0.001	3.44		3.06		2.25	40	72	89
	Residuals	55											
	Relative	4	1.2184	2.1463	<0.001	3.26		3.01		1.88	40	74	85
	Residuals	55											

Damage treatments: TP, Tomato psyllids; CL, Cabbage looper caterpillars; FAW, Fall Armyworm caterpillars; MD, Mechanical damage.

## Results

We measured the VOC emission of the same individuals before and after damage. Individuals within each damage treatment differentially changed their total VOCs amount: some individuals increased their emission after induction while others decreased it or maintained it compared to its own constitutive emission (Figure 1). Only CL- and FAW-damaged plants significantly changed their total mean VOC amount (Table S1).

### Herbivore and mechanical damage induced differential fold-changes in the absolute concentration of individual VOCs

Eleven VOCs were detected in the constitutive blend of Castlemart tomato plants: 3-hexanol (KI=780), α-pinene (KI=924), o-cymene (KI=960), β-myrcene (KI=983), (+)-4-carene (KI=990), α-phellandrene (KI=993), α-terpinene (KI=1006), *p*-cymene (KI=1014), β-phellandrene (KI=1018), nonanal (KI=1095), and decanal (KI=1195). We did not detect the presence of *o-*cymene and α-terpinene in the blend of plants assigned to CL damage or the presence of α-terpinene in the blend of plants assigned to FAW damage (Figure 2, VOC emission before damage).

**Table 2:** Mean absolute concentrations (arbitrary peak area/30min ± SD) of volatile organic compounds emitted buy non-damaged and damaged Castlemart tomato plants.

**VOCs**	**ND**	**TP**	**CL**	**FAW**	**MD**
3-hexanol	6.07±13.28_a_	2.39±3.11_ab_	0.35±0.32_a_	0.09±0.13_a_	21.81±25.78_b_
α-pinene	1.90±3.86	0.62±0.46	1.18±1.15	0.48±0.56	8.96±10.55
o-cymene	9.62±28.80_a_	0.27±0.55_b_	§	0.03±0.05_a_	16.66±15.73_b_
β-myrcene	0.47±2.45	§	0.20±0.30	0.04±0.04	0.58±1.55
(+)-4-carene	44.52±123.90_a_	3.44±4.41_ab_	26.39±18.44_ab_	0.21±0.21_a_	83.87±57.74_b_
α-phellandrene	10.54±28.85	0.84±1.11	7.95±6.02	0.53±0.21	16.75±15.51
α-terpinene	1.24±4.96	§	§	0.01±0.03	1.81±4.79
*p*-cymene	3.53±8.93_a_	0.25±0.36_ab_	0.71±0.49_ab_	0.28±0.16_a_	7.63±3.95_b_
β-phellandrene	265.79±675.77_b_	23.33±24.06_b_	191.67±125.67_a_	2.63±1.84_b_	465.07±284.68_a_
nonanal	9.60±14.84_a_	2.24±5.03_ab_	0.17±0.49_b_	0.81±0.46_ab_	6.34±6.46_ab_
decanal	34.38±68.03_a_	2.56±5.60_ab_	§	0.72±0.28_ab_	27.07±14.68_b_

Damage agents: TP, Tomato psyllid nymphs (n=9); CL, Cabbage looper caterpillars (n=8); FAW, Fall Armyworm caterpillars (n=6); MD, Mechanical damage (n=7); ND, Non-damaged plants (data of 30 individuals assigned to damage treatments); § VOCs not detected. Different letters indicate significant differences among damaged plants (ANOVA or Kruskal Wallis tests at *P*=<0.05).

After damage, the mean absolute concentration of some VOCs significantly changed according to each damage treatment (Figure 2, VOC emission after damage). Fold-changes of the absolute concentration of VOCs were used to compare the induced response of Castlemart plants to different damage treatments. We found a significant effect of damage treatments over the fold-change of the absolute concentration (Table 1, A). The fold-change in the absolute concentration of (+)-4-carene, *p*-cymene, β-phellandrene, and decanal was significantly different among treatments (Figure 3). For example, in the blend of TP-damaged plants, (+)-4-carene increased their concentration around ten times compared to individuals under other treatments. Also the fold-change in concentration of *p*-cymene and β-phellandrene in the blend of TP-damaged plants was greater compared to the other treatments. Size (Table 2) and shape (Table S3) analysis showed a different pattern of variation among damaged plants and non-damaged individuals.

**Table 3:** Relative concentration (mean ± SD percentage) of volatile organic compounds emitted by Castlemart tomato plants before and after different damage treatments.

	**TP**		**CL**		**FAW**		**MD**	
**VOCs**	**Before**	**After**	**Before**	**After**	**Before**	**After**	**Before**	**After**
3-hexanol	6.54±9.98	11.63±18.33	0.13±0.14	0.20±0.20	2.44 ±2.48	1.75±2.36	3.20±3.29	4.94±6.77
α-pinene	1.24±2.61	1.86±0.90	1.45±0.41	**0.41±0.22**	2.79±1.94	9.36±12.11	0.41±0.27	1.71±2.64
o-cymene	0.03±0.10	**0.82±1.01**	0.00±0.00	0.00±0.00	0.41±0.49	1.03±2.24	2.17±1.46	2.10±1.06
β-myrcene	0.00±0.00	0.12±0.35	0.25±0.28	0.06±0.10	0.22±0.39	0.75±0.88	0.09±0.24	0.05±0.13
(+)-4-carene	0.77±1.76	**7.64±5.12**	8.68±2.52	**11.02±1.69**	6.47±4.36	2.90±2.32	10.85±3.05	12.00±2.12
α-phellandrene	0.03±0.09	**2.11±1.30**	0.00±0.00	**3.24±0.56**	5.15±2.02	9.58±2.28	2.43±0.97	2.35±0.99
α-terpinene	0.00±0.00	0.06±0.18	0.00±0.00	0.00±0.00	0.00±0.00	0.14±0.25	0.18±0.32	0.15±0.40
*p*-cymene	0.67±1.11	**0.64±0.68**	2.05±0.67	**0.35±0.18**	1.26±0.75	**5.32±2.91**	0.91±0.27	1.34±0.84
β-phellandrene	37.84±36.23	63.00±21.86	71.59±14.49	**84.63±2.52**	50.69±29.71	40.28±17.06	69.43±6.26	69.95±10.14
nonanal	19.10±14.72	5.95±10.65	0.40±0.85	0.06±0.18	12.86±13.98	14.68±7.01	1.64±1.47	1.00±0.94
decanal	33.77±30.35	**6.72±12.23**	0.16±0.44	0.00±0.00	17.71±20.90	14.20±7.65	8.46±7.43	4.41±1.19

The relative concentration represents the percentage of each compound in a blend relative to the sum of the absolute concentration of all compounds in that blend. Damage treatments: TP, tomato psyllids (n=9); CL, Cabbage looper caterpillars (n=8); FAW, Fall armyworm caterpillars (n=6); MD, Mechanical damage (n=7). Bold numbers indicate significant differences in the mean relative emission compared after damage (paired-*t* test or Wilcoxon test at *P*=<0.05).

**Figure 4:**
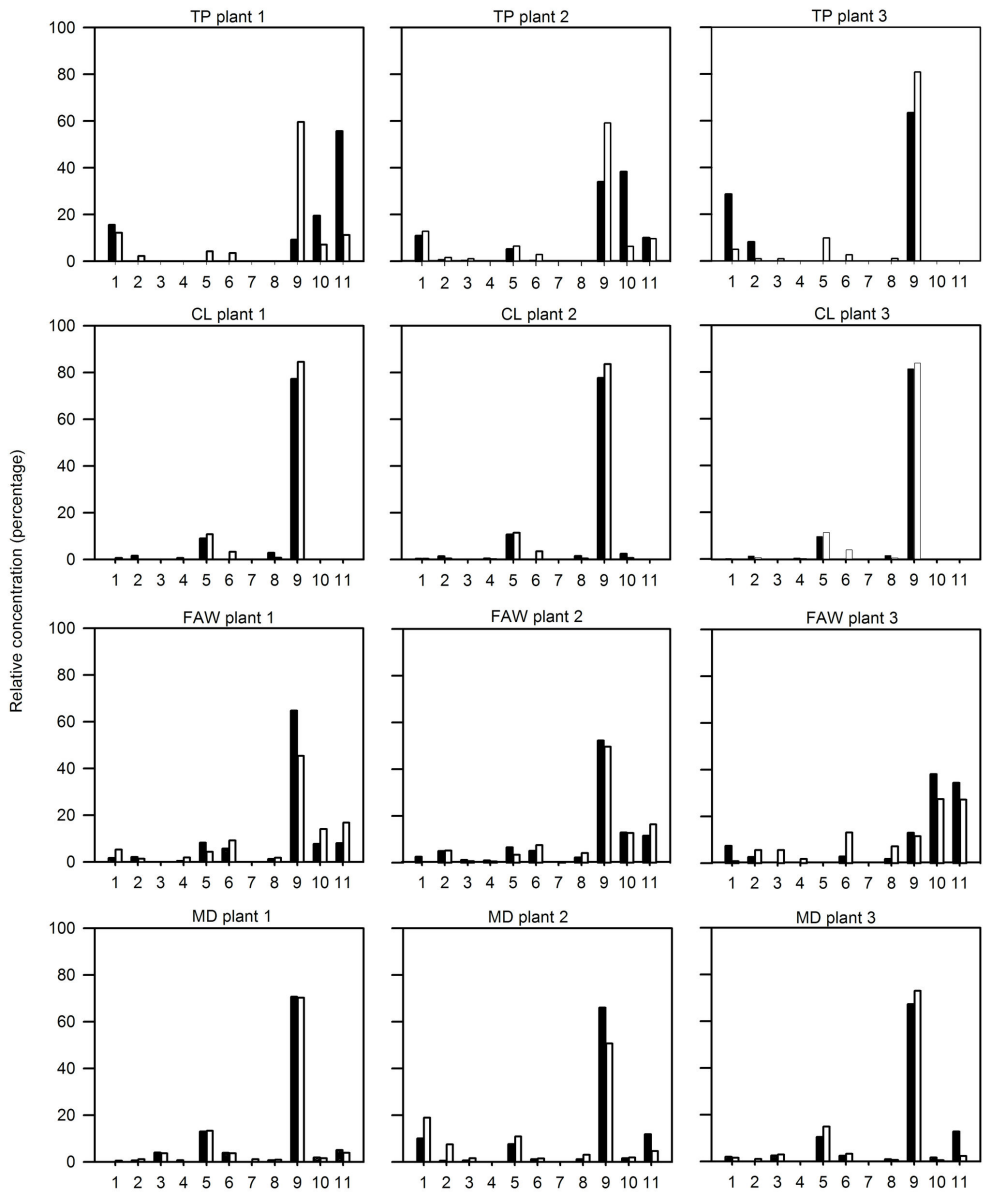
Volatile organic compound blend profile (relative concentration) emitted by three selected tomato individuals before (black bars) and after (white bars) different damage treatments. Footnote of Figure 4. Bars represent the concentration percent of each compound in a blend relative to the sum of the absolute concentration of all compounds in that blend. VOCs: 3-hexanol (1), α-pinene (2), o-cymene (3), β-myrcene (4), (+)-4-carene (5), α-phellandrene (6), α-terpinene (7), *p*-cymene (8), β-phellandrene (9), nonanal (10), and decanal (11)..

The relative concentration of individual VOCs resulted in significant differences before and after damage (paired *t* or Wilcoxon test at *P*=<0.05) mainly in the blend of TP- and CL- damaged plants (Table 3). Changes in the relative concentration varied among individuals within treatments before and after VOC collections. The blend of tomato plants before and after TP feeding was variable in terms of the percentage of a given VOC in the blend (e.g. β-phellandrene, nonanal, and decanal in the blend of TP-damaged plants, Figure 4).

**Figure 5:**
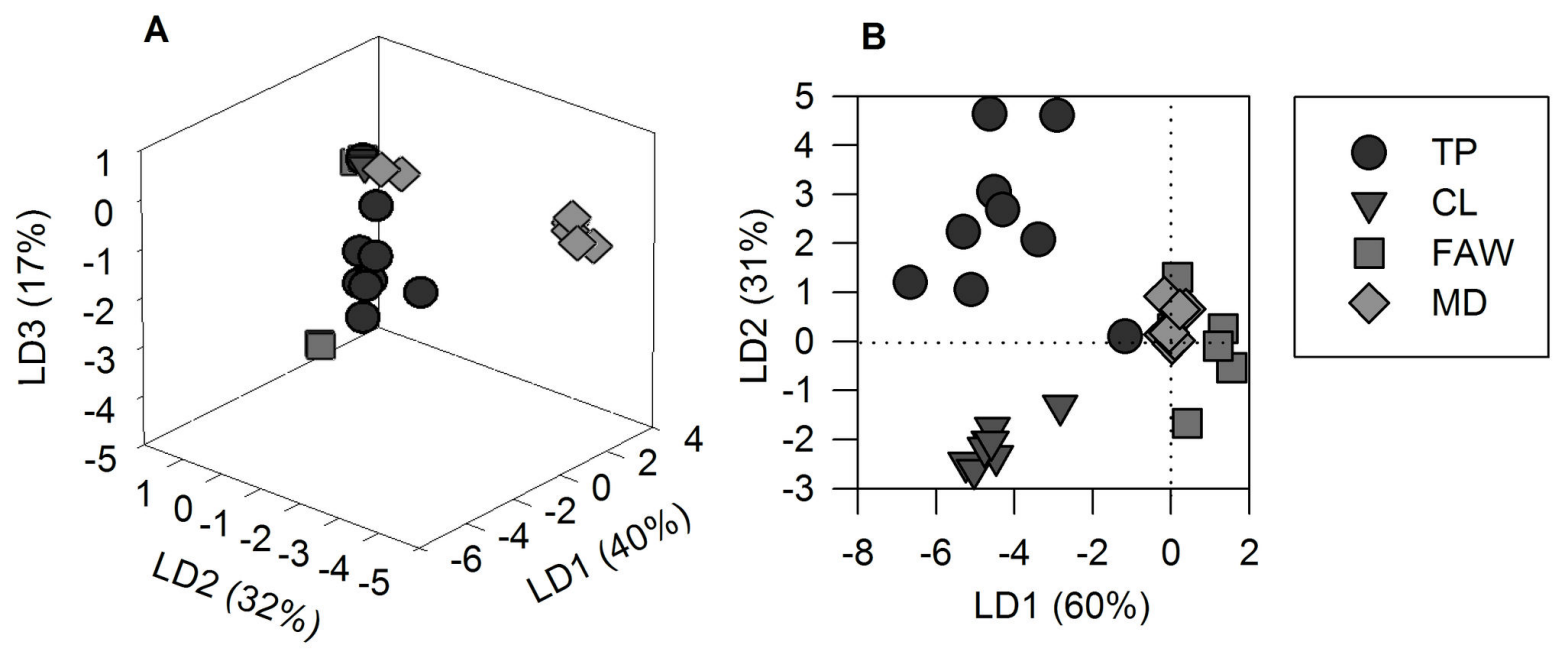
Linear discriminant plots of the fold-change from the constitutive to the induced emission of the absolute (A) and relative (B) volatile organic compounds concentration emitted by individual tomato plants damaged by different agents. Footnote of Figure 5. Each geometrical figure represents an individual. Linear discriminant functions account for 89% of the variability in the absolute concentration analysis and 91% in the relative concentration analysis. Fold-change represents the transition of individuals from a constitutive to an induced state under each damage treatment. Damage treatments: TP, Tomato psyllid nymphs; CL, Cabbage looper caterpillars; FAW, Fall Armyworm caterpillars; MD, Mechanical damage.

### Herbivore and mechanical damage induced different uniformity in odor among individuals

**Figure 6:**
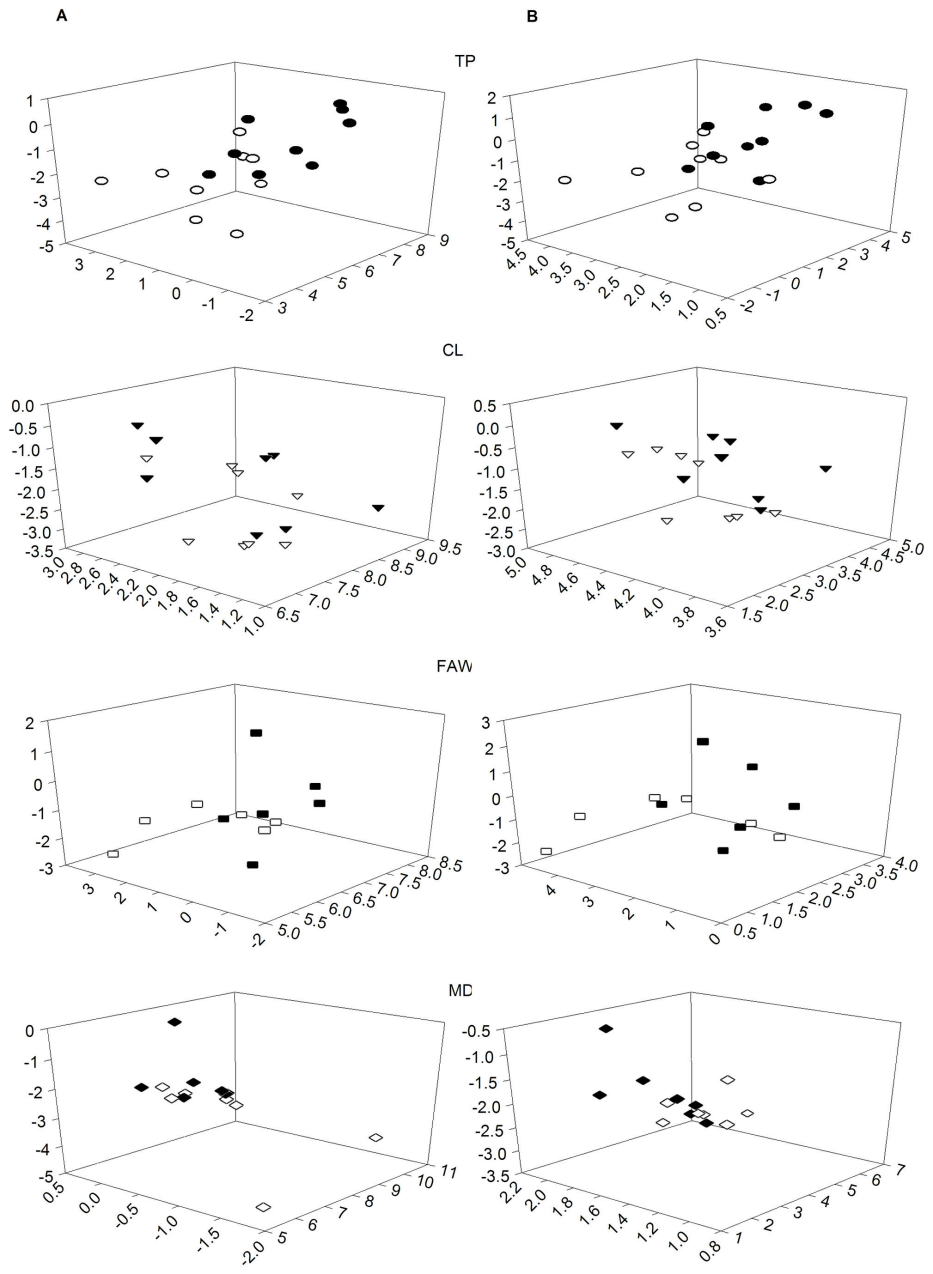
Linear discriminant analysis of the absolute (A) and relative (B) concentration of non-damaged (black points) and damaged (white points) Castlemart tomato individuals. Footnote of Figure 5. Data of non-damaged individuals (30 plants assigned to each treatment) was pooled for comparison with the induced VOC concentration by each treatment. For visual purposes, we extracted the coordinates of individuals before and after each type of damage. Thus, each plot represents the discrimination of damaged individuals against themselves before and after damage. The first three linear discriminant of the absolute and relative VOC concentration accounted for at least 90% of the variation. Damage treatments: TP, Tomato psyllid nypmhs; CL, Cabbage looper caterpillars; FAW, Fall Armyworm caterpillars; MD, mechanical damage.

We calculated the variation at the whole blend level among individuals within treatments based on LDA of the fold-change in concentration and size and shape analysis (Table 1, B LDA). We found differences in the mean values of Euclidian distances among individuals calculated from LD coefficients in fold-change (Figure 5) and size and shape (Figure 6) analyses (Table S4). The blend of CL-damaged plants changed their absolute and relative concentration homogeneously; they had the lowest mean Euclidian distances among individuals compared to other treatments. In terms of the fold-change of the relative concentration, the variation among individuals was similar in damage treatments that removed plant tissue (Table 4, A).

**Table 4:** Similitude (Euclidian distance mean ± SD) among individuals subjected to the same damage treatment according to fold-change (A) and size and shape analysis (B) of volatile organic compounds emitted by Castlemart tomato plants.

**Analysis**	**Concentration**	**ND†**	**TP**	**CL**	**FAW**	**MD**
A. Fold-change	Absolute		2.04±1.45_b_	0.14±0.16_a_	2.36±2.20_b_	2.29±1.85_b_
	Relative		2.80±1.39_a_	0.92±0.77_b_	1.54±0.68_b_	0.54±0.27_b_
B. Size and Shape	Absolute	2.37±0.86_b_	2.44±0.78_b_	1.32±0.60_a_	2.38±1.05_b_	2.36±1.41_b_
	Relative	2.44±0.91_b_	2.48±0.85_b_	1.19±0.53_a_	2.50±1.02_b_	1.87±1.33_ab_

Euclidian distances were calculated from coordinates of linear discriminate analysis of the volatile organic compounds. Damage treatments: TP, Tomato psyllid nymphs; CL, Cabbage looper caterpillar; FAW, Fall Armyworm caterpillar; MD, Mechanical damage. Different letters indicate significant differences among treatments within the same row. † Data from 30 tomato individuals before damage was pooled to calculate the phenotypic distances of the control group in size and shape analysis. Different letters indicate significant differences among treatments.

Individuals were plotted before and after damage according to each treatment using the LDA coefficients that resulted from size and shape analysis (Figure 6). The phenotypic distances calculated from LD coordinates of size and shape analysis showed that CL-damaged individuals had the lowest phenotypic distances among them in the absolute and relative concentration compared to non-damaged individuals. In contrast, TP-damaged plants induced the highest variation among individuals compared to other treatments (Table 4, B).

## Discussion

This study broadens current knowledge of herbivore-specific plant responses. Tomato individuals differentially changed their VOC composition under each treatment in line with herbivore-specific responses previously found in tomato [9-12]. However, we demonstrate that plants differentially respond to herbivore or mechanical damage modifying the fold-change in concentration of specific VOCs in the blend and the uniformity of the VOC emission among individual plants. Euclidian distances calculated from linear discriminant analysis showed that this uniformity in odor is the result of homogeneous or heterogeneous changes in the VOC concentration.

### Herbivore-specific blends and uniformity in odor among damaged individuals

Variation in the components of the constitutive blend of VOCs in Castlemart plants was not unexpected, as genetically based variation in other secondary metabolites has previously been reported [21,22]. After being subjected to different damage treatments, individuals differentially changed the total mean VOC concentration and that one of individual VOCs in their blend. In some cases, the fold-change of some VOCs increased more than ten times relative to its own constitutive emission, suggesting an intense response of plants to that type of damage (e.g. (+)-4-carene and α-phellandrene in the blend of TP-damaged plants). The fact that not all VOCs in the blend changed with the same intensity may indicate that plants respond to the genetic and biochemical changes that herbivores induce (i.e. up- or down-regulation of genes [23] associated to herbivore- or damage-associated molecular patterns [24]). In addition, it is possible that the constitutive concentration plays a role in regulating how many times the concentration of a single VOC can change. If the induced absolute concentration is negatively correlated to the constitutive concentration then there would be a trade-off [6] associated to herbivore-induced responses. To our knowledge there is no information about trade-offs induced by herbivore identity. In addition, insect density and duration of herbivory or mechanical damage can also increase the concentration of VOCs in the blend [14].

Miresmailli et al. [25] showed that plants with moderate herbivory (one larvae per plant) maintained the concentration of some VOCs lower than that of control plants; then, the concentration of VOCs increased with larvae density. We found that the absolute concentration of β*-*phellandrene in the blend of CL-damaged plants was under that of non-damaged individuals (Table 3). Before and after comparison showed that the concentration of β*-*phellandrene increased the most after CL feeding, significantly changing its concentration. This indicates that damage caused by moderate herbivory or a single mechanical damage event was enough to cause specific changes in the absolute concentration of some VOCs that also modified the blend composition (i.e. the relative concentration of VOCs). The lack of significant changes in the absolute and relative VOC concentration in the blend of mechanically-damaged plants may be explained by a unique event of damage that was not long enough to induce changes in the blend. This is in line with other studies showing that only continuous mechanical damage can induce herbivore-like VOC emissions [15]. Although we did not measure plant tissue area removed by the studied insects (which would have been incomparable with TP feeding), we demonstrate that early infestation events can induced changes in odor. In addition, our results show that herbivores differentially changed the uniformity of herbivore-induced VOCs.

The uniformity in odor analyzed in terms of the absolute and relative VOC concentration allows comparing the phenotypic variation induced by herbivores or mechanical damage. Uniform changes in terms of the absolute concentration indicate that individuals within a treatment emit VOCs in the same intensity. In contrast, uniformity in the relative concentration indicates that individuals have similar odors. From size and shape analyzes, we showed that CL induced a uniform VOC emission profile among individuals (i.e. lower Euclidian distance values) compared to individuals under other damage treatments and non-damaged individuals. The same uniformity pattern was found from fold-change in concentration analysis indicating that this uniformity is the result of homogeneous changes in concentration. However, individual plants change their blends’ relative concentration in a similar manner in response to tissue damage agents. In the case of CL-damaged individuals, the fold-change in the absolute and relative VOC concentration was similar producing a uniform induced odor. This pattern indicates that the uniformity in VOC emissions among individuals within a group is also herbivore-specific. Additionally, responses to tissue removal suggest the induction of a regulation process resulting in uniform odors among individuals.

### Ecological implications of herbivore-specific changes in concentration and uniformity in odor

In the first stages of infestation when herbivory can be moderate or intermittent, plants respond to insect feeding or walking [16]. We demonstrated that moderate herbivory induced herbivore-specific VOC blends and affects the uniformity in odor among individuals. In the ecological context, the emission of herbivore-induced VOCs regulates plant-plant interaction [8] and plant-carnivore insects’ interactions [26]. In plant-plant interactions, green-leaf volatiles (GLV) change their concentration in response to mechanical damage which can be a good indicator for future attack priming [1,27]. According to our results, the fold-change in concentration of the GLV 3-hexanol was not significantly different neither within (before and after damage) nor among treatments (in the blends of plants subjected to different treatments). In contrast, the concentration of some monoterpenes changed in the blends emitted by plants within and among treatments (e.g. α-phellandrene). Interestingly, CL and TP feeding induced several changes in the VOC blends of tomato individuals while FAW (at least for the fold-change in the absolute concentration) and mechanical damage did not induce any significant changes. It is possible that moderate herbivory applied to tomato plants under FAW treatment may have not been sufficient to induce more significant changes in the concentration of 3-hexanol or in that of other VOCs. As cited above, Miresmailli et al. [25] showed that the concentration can change with increasing insect density.

In terms of plant-carnivore insects’ interaction, changes in the absolute concentration of single VOCs in the blend may induce behavioral responses in carnivore insects, independently of the presence or change of other volatiles in the blend (e.g. (Z)-jasmone [28]). However, changes in the relative concentration modify the entire blend; in this context, the uniformity in odor among individuals became relevant for carnivore insects that use the whole blend of herbivore-induced VOCs [29]. Thus, the uniformity of the VOC emission among individuals attacked by the same herbivore may determine the interaction with insects that use these compounds to find their hosts [16,30,31].

In conclusion, by including individual constitutive variation, we found that herbivores induce specific changes in the concentration of VOCs in the blend of damaged tomato individuals modifying the uniformity in odor among them. Thus, herbivores induce different levels of phenotypic variation that may affect plant-plant or plant-carnivore insects’ interactions. Further investigation will focus on testing the effects of the uniformity in odor on plant-biotic interactions.

## Supporting Information

Table S1 **Mean total amount (± SD of arbitrary units of peak area) before and after different damage treatments.**
(DOCX)

Table S2 **Absolute concentration fold-change from the constitutive to the induced volatile organic compound emission of each plant plotted in Figure 3.**
(DOCX)

Table S3 **Mean relative concentrations (percentage ± SD) of volatile organic compounds emitted by non-damaged and damaged Castlemart tomato plants.**
(DOCX)

Table S4 **Coefficients of linear discriminants of fold-change and size and shape concentration linear discriminant analysis of Castlemart tomato plants under different damage treatments.**
(DOCX)
