# Supplementary material for: Odor Uniformity among Tomato Individuals in Response to Herbivore Depends on Insect Species
Source: PLoS One. 2013 Oct 9;8(10):e77199. doi: 10.1371/journal.pone.0077199 (PMC3793962; doi:10.1371/journal.pone.0077199)
Supplement: Table S1 — Mean total amount (± SD of arbitrary units of peak area) before and after different damage treatments. (DOCX) [file pone.0077199.s001.docx]

**Table S1** Mean total amount (± SD of arbitrary units of peak area) before and after different damage treatments.

| **Damage** | **VOC emission** | |  |
| --- | --- | --- | --- |
| **treatments** | **Before damage** | **After damage** | ***P*** |
| TP | 66.05±65.69 | 36.00±29.54 | N.S. |
| CL | 60.27±32.07 | 228.64±151.71 | *** |
| FAW | 33.97±27.51 | 5.82±2.83 | *** |
| MD | 1488.01±1542.03 | 660.31±379.60 | N.S. |

Damage treatments: TP, Tomato psyllids; CL, Cabbage looper caterpillars; FAW, Fall Armyworm caterpillars; MD, Mechanical damage. Asterisks indicate significant differences before and after damaged treatment (paired Wilcoxon or *t*-test). *P*=<0.05,*; *P*=<0.01,**; *P*=<0.001, ***. N.S., Non-significant.
