# Supplementary material for: Odor Uniformity among Tomato Individuals in Response to Herbivore Depends on Insect Species
Source: PLoS One. 2013 Oct 9;8(10):e77199. doi: 10.1371/journal.pone.0077199 (PMC3793962; doi:10.1371/journal.pone.0077199)
Supplement: Table S2 — Absolute concentration fold-change from the constitutive to the induced volatile organic compound emission of each plant plotted in Figure 3. (DOCX) [file pone.0077199.s002.docx]

**Table S2** Absolute concentration fold-change from the constitutive to the induced volatile organic compound emission of each plant plotted in Figure 3.

|  | **Volatile organic compounds** | | | |
| --- | --- | --- | --- | --- |
| **Plants** | **(+)-4-carene** | ***p-*cymene** | **β-phellandrene** | **decanal** |
| TP plant 1 | 10.05 | 0.00 | 2.34 | -0.44 |
| TP plant 2 | 0.48 | -6.06e-4 | 0.96 | 8.29e-4 |
| TP plant 3 | 10.65 | 8.35 | 1.92 | 0.00 |
| TP plant 4 | 10.89 | 0.00 | 1.80 | -1.59e-3 |
| TP plant 5 | 4.24 | 2.51 | 3.02 | -0.66 |
| TP plant 6 | 10.74 | 2.31 | 3.93 | 0.43 |
| TP plant 7 | 0.00 | 7.47 | 0.30 | -1.96e-6 |
| TP plant 8 | 11.84 | -7.46e-5 | -0.88 | -1.22e-5 |
| TP plant 9 | 10.02 | -0.06 | 0.49 | -2.46e-3 |
| CL plant 1 | -0.99 | -0.14 | -0.86 | 0.00 |
| CL plant 2 | -1.01 | -0.16 | -1.02 | 0.00 |
| CL plant 3 | 0.56 | -0.36 | 0.99 | 0.00 |
| CL plant 4 | -0.75 | -0.05 | -0.97 | -3.12e-5 |
| CL plant 5 | 0.11 | -0.09 | 0.14 | 0.00 |
| CL plant 6 | -1.30 | -0.10 | -0.99 | 0.00 |
| CL plant 7 | -1.52 | -0.15 | -1.22 | 0.00 |
| CL plant 8 | 1.31 | -0.22 | 0.84 | 0.00 |
| FAW plant 1 | 1.04 | -1.33 | -0.10 | -0.85 |
| FAW plant 2 | 0.63 | -0.79 | 0.46 | -0.31 |
| FAW plant 3 | 9.63 | 1.60 | 1.34 | -0.14 |
| FAW plant 4 | -0.44 | -1.57 | -0.51 | -1.07e-4 |
| FAW plant 5 | -1.45 | 0.73 | -1.34 | 10.85 |
| FAW plant 6 | -1.45 | -2.32e-5 | -0.16 | -1.76e-7 |
| MD plant 1 | -1.03 | -1.69 | -0.96 | -0.66 |
| MD plant 2 | 1.01 | 1.79 | 3.73e-3 | 3.59 |
| MD plant 3 | 0.64 | 1.39 | 0.19 | -0.46 |
| MD plant 4 | -0.21 | -0.29 | -0.27 | -0.29 |
| MD plant 5 | -0.18 | -0.27 | -0.21 | -0.28 |
| MD plant 6 | -1.94 | -3.14 | -2.69 | -0.18 |
| MD plant 7 | 0.26 | 0.84 | 0.45 | 3.25 |

TP, Tomato psyllid; CL, Cabbage looper Caterpillar; FAW, Fall Armyworm; MD, Mechanical damage.
