# Supplementary material for: Odor Uniformity among Tomato Individuals in Response to Herbivore Depends on Insect Species
Source: PLoS One. 2013 Oct 9;8(10):e77199. doi: 10.1371/journal.pone.0077199 (PMC3793962; doi:10.1371/journal.pone.0077199)
Supplement: Table S3 — Mean relative concentrations (percentage ± SD) of volatile organic compounds emitted by non-damaged and damaged Castlemart tomato plants. (DOCX) [file pone.0077199.s003.docx]

**Table S3** Mean relative concentrations (percentage ± SD) of volatile organic compounds emitted by non-damaged and damaged Castlemart tomato plants.

| **VOCs** | **ND** | **TP** | **CL** | **FAW** | **MD** |
| --- | --- | --- | --- | --- | --- |
| 3-hexanol | 3.23±6.08 | 11.63±18.33 | 0.20±0.20 | 1.75±2.36 | 4.94±6.77 |
| α-pinene | 1.41±1.80_ab_ | 1.86±0.90_ab_ | 0.41±0.22_ab_ | 9.36±12.11_a_ | 1.71±2.64_b_ |
| *o*-cymene | 0.60±1.14_a_ | 0.82±1.01_ab_ | § | 1.03±2.24_a_ | 2.10±1.06_a_ |
| β-myrcene | 0.13±0.26_ab_ | § | 0.06±0.10_ab_ | 0.75±0.88_a_ | 0.05±0.13_b_ |
| (+)-4-carene | 6.37±4.86_b_ | 7.64±5.12_ab_ | 11.02±1.69_a_ | 2.90±2.32_b_ | 12.00±2.12_a_ |
| α-phellandrene | 1.60±2.27_a_ | 2.11±1.30_ab_ | 3.24±0.56_b_ | 9.58±2.28_ab_ | 2.35±0.99_ab_ |
| α-terpinene | 0.04±0.17_ab_ | § | § | 0.14±0.25_a_ | 0.15±0.40_b_ |
| *p*-cymene | 1.21±0.93 | 0.64±0.68 | 0.35±0.18 | 5.32±2.91 | 1.34±0.84 |
| β-phellandrene | 56.78±28.13_b_ | 63.00±21.86_ab_ | 84.63±2.52_a_ | 40.28±17.06_c_ | 69.95±10.14_a_ |
| Nonanal | 8.79±12.74_a_ | 5.95±10.65_ab_ | 0.06±0.18_b_ | 14.68±7.01_ab_ | 1.00±0.94_ab_ |
| Decanal | 15.69±22.86_a_ | 6.72±12.23_ab_ | § | 14.20±7.65_ab_ | 4.41±1.19_b_ |

Damage agents: TP, Tomato psyllid nymphs (n=9); CL, Cabbage Looper caterpillars (n=8); FAW, Fall Armyworm caterpillars (n=6); MD, Mechanical damage (n=7); ND, Non-damaged plants (pooled data of 30 individuals before damage treatments); §, VOCs not detected. Different letters indicate significant differences among damaged plants (ANOVA or Kruskal Wallis tests at *P*=<0.05).
