# Supplementary material for: Odor Uniformity among Tomato Individuals in Response to Herbivore Depends on Insect Species
Source: PLoS One. 2013 Oct 9;8(10):e77199. doi: 10.1371/journal.pone.0077199 (PMC3793962; doi:10.1371/journal.pone.0077199)
Supplement: Table S4 — Coefficients of linear discriminants of fold-change and size and shape concentration linear discriminant analysis of Castlemart tomato plants under different damage treatments. (DOCX) [file pone.0077199.s004.docx]

**Table S4** Coefficients of linear discriminants of fold-change and size and shape concentration linear discriminant analysis of Castlemart tomato plants under different damage treatments.

| \|  \| **Fold-change** \| \| \| \| \| **Size and shape** \| \| \| \| \| \| \| --- \| --- \| --- \| --- \| --- \| --- \| --- \| --- \| --- \| --- \| --- \| --- \| \|  \| **Absolute** \| \| \| **Relative** \| \| **Absolute** \| \| \| **Relative** \| \| \| \| **VOC** \| **LD1** \| **LD2** \| **LD3** \| **LD1** \| **LD2** \| **LD1** \| **LD2** \| **LD3** \| **LD1** \| **LD2** \| **LD3** \| \| 1 \| - 0.00003 \| -0.000021 \| -0.000002 \| -0.01 \| 0.00 \| 0.04 \| -0.14 \| -0.12 \| 0.12 \| -0.08 \| -0.08 \| \| 2 \| -0.000048 \| -0.000038 \| -0.000009 \| -0.02 \| 0.05 \| **-0.39** \| 0.07 \| 0.10 \| **-0.40** \| -0.07 \| 0.03 \| \| 3 \| 0.000001 \| 0.000006 \| -0.000019 \| -0.01 \| 0.18 \| -0.02 \| -0.15 \| -0.01 \| 0.00 \| **-0.17** \| -0.11 \| \| 4 \| 0.000256 \| **0.001949** \| **0.000407** \| **0.11** \| **-0.55** \| 0.11 \| -0.07 \| 0.05 \| 0.12 \| -0.04 \| 0.07 \| \| 5 \| -0.000007 \| 0.000009 \| -0.000005 \| -0.01 \| 0.03 \| -0.28 \| -0.05 \| -0.26 \| -0.17 \| -0.06 \| **-0.27** \| \| 6 \| **-0.000163** \| **-0.000184** \| -0.000026 \| -0.13 \| -0.07 \| 0.07 \| 0.15 \| 0.16 \| -0.02 \| 0.13 \| **0.16** \| \| 7 \| **0.000981** \| 0.001126 \| 0.000208 \| 0.04 \| 0.04 \| -0.28 \| -0.11 \| **-0.35** \|  \|  \|  \| \| 8 \| -0.000414 \| -0.000131 \| **-0.000104** \| 0.09 \| 0.08 \| 0.28 \| -0.18 \| 0.09 \| 0.31 \| -0.09 \| **0.16** \| \| 9 \| 0.000008 \| -0.000007 \| 0.000002 \| **-0.21** \| **0.22** \| **0.83** \| **0.21** \| -0.18 \| **0.66** \| **0.41** \| -0.15 \| \| 10 \| -0.000041 \| 0.000042 \| -0.000017 \| -0.02 \| -0.08 \| -0.13 \| 0.17 \| **0.19** \| -0.22 \| 0.08 \| 0.14 \| \| 11 \| 0.000121 \| -0.000101 \| -0.000031 \| 0.04 \| 0.07 \| 0.08 \| **-0.19** \| -0.03 \| 0.14 \| -0.14 \| -0.002 \| |  |  |
| --- | --- | --- | --- | --- | --- | --- | --- | --- | --- | --- | --- | --- | --- | --- | --- | --- | --- | --- | --- | --- | --- | --- | --- | --- | --- | --- | --- | --- | --- | --- | --- | --- | --- | --- | --- | --- | --- | --- | --- | --- | --- | --- | --- | --- | --- | --- | --- | --- | --- | --- | --- | --- | --- | --- | --- | --- | --- | --- | --- | --- | --- | --- | --- | --- | --- | --- | --- | --- | --- | --- | --- | --- | --- | --- | --- | --- | --- | --- | --- | --- | --- | --- | --- | --- | --- | --- | --- | --- | --- | --- | --- | --- | --- | --- | --- | --- | --- | --- | --- | --- | --- | --- | --- | --- | --- | --- | --- | --- | --- | --- | --- | --- | --- | --- | --- | --- | --- | --- | --- | --- | --- | --- | --- | --- | --- | --- | --- | --- | --- | --- | --- | --- | --- | --- | --- | --- | --- | --- | --- | --- | --- | --- | --- | --- | --- | --- | --- | --- | --- | --- | --- | --- | --- | --- | --- | --- | --- | --- | --- | --- | --- | --- | --- | --- | --- | --- | --- | --- | --- | --- |

VOCs: (1) 3-hexanol, (2) α-pinene, (3) *o*-cymene, (4) β-myrcene, (5) (+)-4-carene, (6) α-phellandrene, (7) α-terpinene, (8) *p*-cymene, (9) β-phellandrene, (10) nonanal, and (11) decanal. VOCs with coefficients in bold indicate the strongest contribution to the ordination of the absolute and relative volatile concentration discrimination.
